# Antiapoptotic and antioxidative effects of cerium oxide nanoparticles on the testicular tissues of streptozotocin-induced diabetic rats: An experimental study

**DOI:** 10.18502/ijrm.v19i7.9465

**Published:** 2021-08-16

**Authors:** Torab Solgi, Iraj Amiri, Sara Soleimani Asl, Massoud Saidijam, Banafsheh Mirzaei Seresht, Tayebe Artimani

**Affiliations:** ^1^Department of Anatomy, School of Medicine, Hamadan University of Medical Sciences, Hamadan, Iran.; ^2^Endometrium and Endometriosis Research Center, Hamadan University of Medical Sciences, Hamadan, Iran.; ^3^Research Center for Molecular Medicine, Hamadan University of Medical Sciences, Hamadan, Iran.

**Keywords:** Apoptosis, Nanoceria, Diabetes, Oxidative stress, Testis.

## Abstract

**Background:**

Cerium dioxide nanoparticles (CNPs) due to the antidiabetic and antioxidant activities are proposed for the treatment of oxidative stress-associated diseases.

**Objective:**

To examine the impact of CNPs on hyperglycemia-induced apoptosis and oxidative stress in the testis of diabetic rats.

**Materials and Methods:**

Twenty-four male rats were divided into four groups (n = 6/each) as diabetic rats, CNPs group, diabetic + CNPs rats, and controls. The control group was fed only mouse food and water. Rats became diabetic through receiving streptozotocin (STZ) 60 mg/kg. CNPs were given to the rats at a dose of 30 mg/kg daily for 2 wk. Malondialdehyde and total thiol group (TTG) levels were measured using spectrofluorometer. Expression of b-cell lymphoma protein 2-associated X protein (*BAX*) and b-cell lymphoma protein 2 (*Bcl-2*) were investigated using quantitative real-time polymerase chain reaction. Western blot analysis was used to examine caspase 3 protein levels.

**Results:**

The content of malondialdehyde significantly increased in the STZ-diabetic rats, while TTG levels demonstrated a remarkable decrease. Caspase-3, *BAX*, and *BAX*/*Bcl-2* mRNA ratio raised significantly in the STZ-diabetic rats. On the other hand, *Bcl-2* mRNA levels reduced in the testis of diabetic rats (p = 0.006). Intervention with CNPs caused a substantial increase in the TTG levels, while the malondialdehyde contents, caspase-3, *BAX* levels, as well as *BAX/Bcl-2* mRNA ratio were considerably decreased following CNPs treatment. Administration of CNPs increased mRNA levels of *Bcl-2* (p < 0.0001).

**Conclusion:**

CNPs treatment attenuates testicular apoptosis and oxidative stress induced by diabetes. This nanoparticle might be suggested for the treatment of diabetes-associated reproductive disorders.

## 1. Introduction

Diabetes is associated with the impairment of semen quality, erectile dysfunction, testicular damage, decreased impotence, and fertility potential in men (1). A previous study on diabetes has shown that hyperglycemia may influence male infertility and reproductive complications via activation of oxidative stress and apoptosis (2).

Oxidative stress is described as the overproduction of reactive oxygen species (ROS), which causes damage to DNA and protein, and activates apoptosis process within the cells (3). Excessive production of ROS and enhanced levels of apoptotic cells in the testes of induced diabetic rats result in sperm DNA damage, reduction of sperm count, decrease in the number of Leydig cells and synthesis of testosterone, and subsequently, disturbs the spermatogenesis process (4).

Recent studies have introduced different natural products with remarkable antioxidant and antidiabetic activities (5–8). Cerium dioxide nanoparticles (CNPs), identified as nanoceria, frequently exhibit powerful medicinal effects on diabetes because of their self-regenerative antioxidant activity with minimum toxicity (5). The antioxidant characteristics of CNPs attribute to the electronic configuration at the nanoscale. A high proportion of surface area to the volume as particle size decreases, accompanied with a unique ability of quick and reversible switching between Ce3+ and Ce4+ oxidation states, (6) provide suitable “reactive sites” or “hot spots” for scavenging radicals and ROS (7). CNPs present effective antidiabetic action via modulation of nuclear factor kappa-light-chain-enhancer of activated B cells/nuclear factor erythroid-2-related factor 2 (*NF-κB/Nrf2*) signaling, and significant abrogation of the apoptosis (8, 9). Nanoceria, as a sensor for ROS, can reduce apoptotic cell death and reveals a pro-survival effect (10).

Apart from the antioxidant property, nanoceria exerts pro-oxidant activity which depends on the cell status. Therefore, nanoceria depending on the pH of the redox reaction reveals different activities. Neutral pH enhances cytoprotective effects of CNPs, while under acidic environment (within cancerous cells), CNPs as the producer of H${}_{2}$O${}_{2}$ exhibit cytotoxic properties (11). However, further features such as the form and size of particle, interfaces between the phases, and surface additives or ligands determine the pro-oxidant or antioxidant activity of the nanoceria (12). In the previous work of the authors, CNPs treatment demonstrated a significant reduction in the oxidative stress induced by diabetes through the upregulation of *Nrf2* and its downstream antioxidant genes including heme oxygenase-1, nicotinamide adenine dinucleotide phosphate reduced quinone dehydrogenase 1, and glutamate-cysteine ligase catalytic subunit (13). Moreover, it was shown that CNPs have ameliorative effects on the sperm parameters, sperm DNA fragmentation, and testicular damage. There is no report on the impact of CNPs on the apoptosis and the thiol levels in the testicular tissues of the diabetic rats. Therefore, the present study was designed to examine the antioxidative and antiapoptotic potential of the CNPs as a therapeutic agent on the testicular injuries of the diabetic rats.

## 2. Materials and Methods

### Study design and sample collection

This experimental study was conducted at the Anatomy Department, Embryology Lab of the Faculty of Medicine, Hamadan University of Medical Sciences, Hamadan, Iran during 2019. A total of 24 male Wistar rats (aged 10–12 wk and weighing 250–300 gr) were purchased from the animal house of Hamadan University of Medical Sciences and housed in the cages under the standard conditions at a temperature of 22 ± 1∘C, 45–55% humidity, and a 12-hr light/dark cycle (9, 13). The experimental animals were divided into four groups (n = 6/each) and received treatment as: diabetic rats, CNPs group, diabetic + CNPs rats, and control group. Diabetic model was induced by intraperitoneal injection of 60 mg/kg streptozotocin (STZ, Sigma, St Louis, MO) diabetes mellitus was confirmed via the measurement of blood glucose concentration (Roche Diagnostics, Mannheim, Germany), 72 hr after the STZ injection. Rats with a blood glucose > 300 mg/dl were included in the study and 30 mg/kg/daily of CNPs was interpretably injected (US, Research Nanomaterials, Inc.) for 2 wk. The control group, on the other hand, was only feed with water and standard laboratory food without receiving any treatment. Diabetic rats received STZ injection performed as 30 mg/kg/daily, and the CNPs group were treated with CNPs as 30 mg/kg daily for 2 wk. Rats in the diabetic + CNPs group after the STZ injection were treated with 30 mg/kg of CNPs for 2 wk. Further, after 24 hr of last treatment, rats were anesthetized with chloroform, and then the testes were separated and immediately stored at –80∘C until further use (9, 13).

### Assessment of lipid peroxidation and total thiol molecules

Lipid peroxidation was assessed through the determination of malondialdehyde (MDA). Contents of MDA were measured spectrofluorometrically in the homogenate of testis tissue by thiobarbituric acid-reactive substances using Yagi's methods (14). The results were expressed as 1 mg of protein content in a set of samples.

The total thiol group (TTG) was measured in the testicular tissue homogenate using di-thio-nitro-benzoic acid reagent. The reaction between TTG and 5, 5'-dithiobis-(2-nitrobenzoic acid) resulted in a yellow compound that possessed an excellent absorption in the spectrophotometer at 412 nm.

### Quantitative real-time polymerase chain reaction analysis 

The total RNA was extracted with RNX-Plus${}^{\mathrm{TM}}$ (Cinnagen, Iran), and any genomic contamination was checked using DNase 1 (Fermentas, Vilnius, and Lithuania). First-strand complementary DNA (cDNA) was synthesized from isolated RNA using superscript II and reverse transcribed by oligo (dt) primers (Fermentas, Vilnius, and Lithuania). The expression of b-cell lymphoma protein 2 (*Bcl*-*2*) and Bcl-2-associated X protein (*BAX*) genes were examined by quantitative real-time PCR using specific primers prepared by the Bioneer Corporation under the PCR condition as described previously. Table I shows the characteristics of the primers. Beta-actin was used as an internal reference gene, and PCR analysis was carried out using a 2-ΔΔ CT  method, as explained before (13).

### Western blot analysis

Western blot analysis was performed to measure the protein expression levels of Caspase-3 (15). Briefly, testicular tissues were lysed in a lysis buffer that comprised of Ripa buffer (Sigma Aldrich, Germany) and a protease inhibitor cocktail (Sigma Aldrich, Germany); the resulting mixture was then centrifuged for 20 min at 4∘C and the supernatant was collected and kept until use. The total protein was loaded on sodium dodecyl sulfate-polyacrylamide gel (SDS-PAGE) and then transferred from the gel onto a nitrocellulose membrane to separate protein bands (Amersham Pharmacia Biotech, UK). The membranes were blocked with 5% nonfat dry milk and incubated with Anti-Caspase-3 and β-actin antibodies overnight at 4∘C and horseradish peroxidase (HRP) conjugated secondary antibody for 1 hr. ECL Western Blotting Substrate Kit (Abcam, UK) was used to determine the bands and the densities were measured by the ImageJ software.

**Table 1:** The primer sequences (5'-3') used in qRT-PCR


**Gene**	**Forward primer**	**Reverse primer**	**Accession number**	**Annealing temperature**	**Product size bp**
*BAX*	GGAGACACCTGAGCTGACCTTG	CTGCCACACGGAAGACCTC	NM_017059	55	170
*Bcl-2*	CCGGGAGAACAGGGTATGATA	TCAGGCTGGAAGGAGAAGATGC	NM_016993	54	151
*β-actin*	CGCGAGTACAACCTTCTTGC	ATACCCACCATCACACCCTGG	NM_031144.3	60	200
*BAX:* Bcl-2-associated X prote, Bcl-2: b-cell lymphoma protein 2					

### Ethical considerations

The study was approved by the ethics committee of the Hamadan University of Medical Sciences, Haman, Iran (Code: IR.UMSHA.REC.1396.884). Rats were cared in accordance with the Guide for Care and Use of Laboratory Animals, the National Academic Press.

### Statistical analysis

Data were presented as mean ± SEM and analyzed with Social Sciences statistical package v. 16 (SPSS, Inc., Chicago, IL). One-way ANOVA with Tukey's post hoc test was used to compare the differences between the multiple groups. P < 0.05 was considered statistically significant.

## 3. Results

### Effect of the CNPs treatment on oxidative stress status

**Figure 1:**
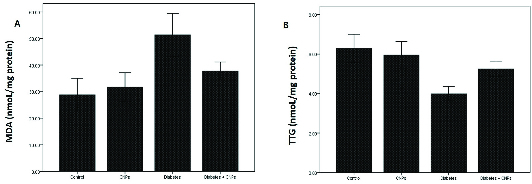
Comparison of malondialdehyde (MDA) and total thiol concentrations group (TTG) concentrations between the groups. Results are presented as Mean ± SD. (A) Comparison of MDA concentration between the groups. There was a significant difference in the concentration of MDA between the control and diabetes groups (p = 0.001). Concentration of MDA demonstrated significant increase in the cerium dioxide nanoparticles (CNPs) + diabetes group when compared to the diabetes group (p = 0.032). (B) Comparison of TTG levels between the groups. TTG levels reduced significantly in the diabetes group in comparison to the control group (p < 0.0001). Following the treatment with CNPs, the TTG levels increased significantly (p = 0.034).

To assess the impact of CNPs treatment on the oxidative stress status, a concentration of MDA (a biomarker of oxidative stress) and TTG (a significant portion of the total body antioxidants) were evaluated in the rat testicular tissue of the different groups (Figure 1). The concentration of MDA revealed a significant increase in diabetic rats (51.4 ± 4.04 nmol/mg protein) when compared to the control group (p = 0.001). Following the CPNs administration, the MDA content reduced remarkably in the diabetic rats (p = 0.032, Figure 1A). Moreover, the TTG concentration in the diabetic group showed a notable decrease in comparison to the control group (p < 0.0001). When CNPs were administered to the diabetic + CNPs group, a considerable enhancement in the concentration of TTG was noticed (p = 0.034, Figure 1B).

### Effect of CNPs on *Bcl-2* and *BAX* gene expression and *BAX/Bcl-2* mRNA ratio

**Figure 2:**
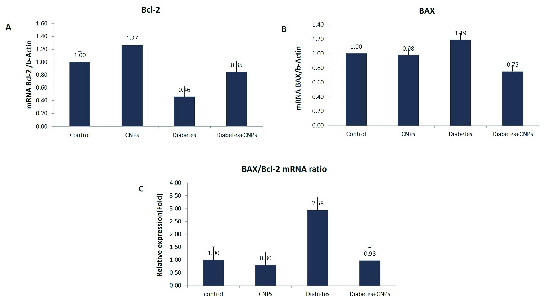
B-cell lymphoma protein 2 (*Bcl-2*) and BCL2-associated X protein (*BAX*) mRNA expression and *BAX/Bcl-2* mRNA ratio in the testicular tissue of experimental groups. Results are presented as Mean ± SE. (A) Expression of *Bcl-2 *compared between the groups. The results revealed signifcant differences between the control and diabetic groups (p = 0.006), diabetic and cerium dioxide nanoparticles (CNPs) groups (p = 0.001), and between diabetic and diabetic + CNPs groups (p < 0.0001). (B) Expression of *BAX *compared between the groups. There were significant differences in the expression levels of *BAX *between the control and diabetic groups (p = 0.01), diabetic and diabetic + CNPs groups (p = 0.01), and between control and diabetic + CNPs groups (p = 0.01). (C) Comparison of *BAX/Bcl-2* mRNA ratio between the groups. Significant differences were observed between the control and diabetic groups (p < 0.0001), between diabetic and CNPs groups (p < 0.0001), diabetic and diabetic + CNPs groups (p < 0.0001), and between control and diabetic + CNPs groups (p < 0.0001).

Figure 2 shows the mRNA expression levels of *Bcl-2* and *BAX*, and *BAX/Bcl-2* mRNA ratio in the testicular tissue of the experimental groups. As shown in Figure 2A, diabetes caused a considerable decrease in the *Bcl-2* gene expression of STZ-induced diabetic rats when compared to the control group (p = 0.006). Administration the administration of CNPs to the diabetic rats upregulated the mRNA expression levels of *Bcl-2* in the testicular tissue (p < 0.0001). Moreover, *BAX* gene expression was higher in the diabetic rats compared to the control group (p = 0.01). Following the CNPs injection to the diabetic rats, the pro-poptotic *BAX* gene was downregulated in the testicular tissue samples (p = 0.01, Figure 2B). Furthermore, the *BAX/Bcl-2* mRNA ratio in the diabetic rat was remarkably higher than controls (p < 0.0001). CNPs treatment of the diabetic rats resulted in a noticeable decline in the *BAX/Bcl-2* mRNA ratio (p < 0.0001, Figure 2C).

### WB analysis of Caspase-3 expression

**Figure 3:**
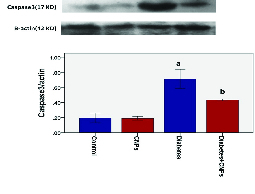
The effect of cerium dioxide nanoparticles (CNPs) on Caspase-3 protein concentration in the testicular tissue of experimental groups. Results are presented as Mean ± SE. (a) Difference between the control and CNPs with diabetic groups (p = 0.001). (b) Difference between the diabetic and diabetic + CNPs groups (p = 0.04).

The WB analysis showed a specific reaction between Anti-Caspase-3 antibody and 17 KD protein bands (Figure 3). Quantification of bands revealed a higher expression of Caspase-3 in the diabetic group in comparison to the control and CNPs groups (p < 0.001). As shown in Figure 3, a significant difference was noted in the expression of Caspase-3 between the diabetic group and the diabetic + CNPs group (p = 0.04).

## 4. Discussion

In this study, the impact of CNPs treatment was evaluated on the apoptosis and oxidative stress status in the testicular tissue of the STZ-induced diabetic rats. Our findings releveled that nanoceria has protective effects against hyperglycemia-induced oxidative stress and apoptosis of the testicular cells. Nanoparticles, due to the antioxidant auto-regenerative ability and low toxicity, can reduce oxidative stress and improve microcirculation in many oxidative stress-related diseases such as diabetes (16). Moreover, hyperglycemia-induced oxidative stress can shift the balance between the antioxidant and oxidant status in favor of oxidants, which may damage the DNA of the germ cells and subsequently lead to subfertility or even infertility (16, 17). Lipid peroxidation is a critical biomarker of hyperglycemia-induced oxidative stress (18). MDA is the secondary product of lipid peroxidation and the most popular and substantial index of oxidative stress. The quantification of MDA content in the testicular tissue is a useful determinant for the grading of oxidative stress injuries (19).

Previous reports demonstrated a significant association between the diabetic testicular damage and enhanced MDA levels as an index of lipid peroxidation, and reduction of glutathione levels and superoxide dismutase in the testicular tissues (20, 21). In agreement with these studies, the present study revealed elevated levels of MDA and reduced concentration of TTG in the testicular tissues of the diabetic rats.

The free radicals through the alteration of thiol groups cause changes in the protein function and structure (18). Thiols due to the potent antioxidant activity are capable of protecting cells against the free radicals-induced tissue damage.

In recent years, several studies have investigated the impact of CNPs on the treatment of various disorders associated with the oxidative stress and inflammation (22–24). CNPs exhibit antioxidative and antiapoptotic properties on the hepatocytes, cardiomyocytes, neuronal culture, and endothelial cells (23, 25).

In the previous work of the authors, following the CNPs treatment on the testes of the diabetic rats, a marked increase in the total antioxidant capacity levels and a considerable decrease in the total oxidant status concentrations were observed, illustrating a significant protection against oxidative stress (13). Furthermore, the present work showed that intervention with CNPs attenuates MDA levels and enhances TTG contents in the testicular tissue of the diabetic rats, implying antioxidative activity of this nanoparticle by reducing destructive free radicals and strengthening the antioxidant defense.

ROS can also indirectly activate apoptosis via changes in the intracellular redox balance of cells and depletion of glutathione. Proapoptotic *Bcl-2* family, such as *BAX* proteins located in mitochondria, have a critical role in the regulation of apoptosis. While *BAX* protein induces cell death, *Bcl-2* inhibits it. Furthermore, *BAX* displays ion channel activity and releases cytochrome c, which involves the initiation of the intrinsic apoptosis pathway (18).

*Bcl-2* contributes to the regulation of antioxidant pathway or protection of cells against lipid oxidation without changing the intracellular ROS levels, hence indirectly exhibiting the potent antioxidant effects. Moreover, the *BAX/Bcl-2* ratio has been regarded as an index to determine the fate of the cells, whether to survive or die (19).

Furthermore, previous works highlighted that hyperglycemia induces oxidative stress and testicular apoptosis and activates p38 mitogen-activated protein kinase and p53-signaling pathways that are associated with the mitochondria–cell death cascade and elevation in the *BAX/Bcl-2* ratio (24, 26).

The current study showed that hyperglycemia results in a significant upregulation of the proapoptotic *BAX* gene and downregulation of the antiapoptotic *Bcl-2* gene, as well as increased *BAX/Bcl-2* ratio in the testes of the diabetic rats. These changes were associated with the higher lipid peroxidation, suggesting that ROS induces apoptosis of the testicular cells.

The present study showed that CNPs treatment suppresses diabetes-induced apoptosis in the testicular tissue by reducing the proapoptotic *BAX* gene expression and enhancing antiapoptotic *Bcl-2* gene expression along with the downregulation of *BAX/Bcl-2* ratio. The results of the present study are in line with those reported earlier, which it has been indicated that cerium and yttrium oxide nanoparticles suppress apoptosis and peroxidation of lipid in high glucose-treated undifferentiated PC12 cells via regulation of *Bax*, *Bcl-2* and Caspase-3 apoptotic proteins and lowering ROS levels (18).

Caspases are a class of proteases that contribute to the apoptosis cascade by cleaving intracellular targets. Among the caspases, Caspase-3 has a crucial role in the disturbance of mitochondrial function, and its activation is the final step of the apoptosis process. Therefore, altered levels of Caspase-3 is a reliable marker of apoptosis (27).

The results of the present study showed a remarkable enhancement of Caspase-3 levels in the testicular tissue of the diabetic group, and CNPs treatment was capable of decreasing the elevated levels. The regulation of this protein by CNPs implies the antiapoptotic property of these nanoparticles.

The underlying mechanisms for the antiapoptotic effects of CNPs are still unclear. It was reported that CNPs can enhance mitochondrial membrane potential and inhibit mitochondrial dysfunction caused by proapoptotic factors (25). ROS cause releasing of Ca2${}^{+}$ from the endoplasmic reticulum and mitochondria, which activates the mitochondrial Ca2${}^{+}$ uptake (28). It has been shown that nanoceria by blocking the Ca2${}^{+}$ channels attenuates mitochondrial depolarization and apoptosis (29). Nanoceria scavenges ROS through the cycling between Ce3${}^{+}$ and Ce4${}^{+}$ states and mimics superoxide dismutase and catalase activities (30). Reduction of DNA fraction and decline in the ratio of necrosis and apoptosis, as well as enhancement of mitochondrial membrane potential, indicate apoptosis alleviation induced by ROS. These findings demonstrate that nanoceria is a novel nanoparticle with a strong ability to prevent oxidative stress injuries to the testicular tissue, which can be used as a therapeutic agent to control diabetes complications associated with the reproductive disorders (25).

## 5. Conclusion

In the present study, STZ-induced diabetic rats exhibited elevation of oxidative stress identified by higher levels of MDA and lower TTG levels. Moreover, STZ caused a significant rise in the apoptotic cells associated with the upregulation of proapoptotic *BAX* and downregulation of antiapoptotic *Bcl-2* genes, and higher expression of Caspase-3 in the testes. CNPs administration considerably reversed the hyperglycemia-induced detrimental effects. These data suggest that the CNPs possess therapeutic potential for the treatment of diabetes-associated reproductive disorders.

##  Conflict of Interest 

The authors declare that they have no conflict of interest.
